# Immunostimulatory Activity of the Cytokine-Based Biologic, IRX-2, on Human Papillomavirus-Exposed Langerhans Cells

**DOI:** 10.1089/jir.2015.0115

**Published:** 2016-05-01

**Authors:** Diane M. Da Silva, Andrew W. Woodham, Paul H. Naylor, James E. Egan, Neil L. Berinstein, W. Martin Kast

**Affiliations:** ^1^ Department of Obstetrics & Gynecology, University of Southern California, Los Angeles, California.; ^2^ Norris Comprehensive Cancer Center, University of Southern California, Los Angeles, California.; ^3^ Department of Molecular Microbiology & Immunology, University of Southern California, Los Angeles, California.; ^4^ Department of Internal Medicine, Wayne State University School of Medicine, Detroit, Michigan.; ^5^ IRX Therapeutics, Inc., New York, New York.

## Abstract

Langerhans cells (LCs) are the antigen-presenting cells of the epithelial layer and are responsible for initiating immune responses against skin and mucosa-invading viruses. Human papillomavirus (HPV)-mediated suppression of LC function is a crucial mechanism of HPV immune evasion, which can lead to persistent infection and development of several human cancers, including cervical, anal, and head and neck cancers. The cell-derived cytokine-based biologic, IRX-2, consists of multiple well-defined cytokines and is broadly active on various immune cell subsets. In this study, we investigated primary human LC activation after exposure to HPV16, followed by treatment with IRX-2 *in vitro*, and evaluated their subsequent ability to induce HPV16-specific T cells. In contrast to its activity on dendritic cells, HPV16 alone is not sufficient to induce phenotypic and functional activation of LCs. However, IRX-2 induces a significant upregulation of antigen presentation and costimulatory molecules, T helper 1 (Th1)-associated cytokine release, and chemokine-directed migration of LCs pre-exposed to HPV16. Furthermore, LCs treated with IRX-2 after HPV16 exposure induced CD8^+^ T-cell responses against specific HLA-A*0201-binding HPV16 T-cell epitopes. The present study suggests that IRX-2 is an attractive immunomodulator for assisting the immune response in eradication of HPV-infected cells, thereby potentially preventing HPV-induced cancers.

## Introduction

Persistent high-risk human papillomavirus (hrHPV) infection leads to the development of numerous human cancers, including cervical, vaginal, vulvar, anal, and an increasing proportion of head and neck cancers, which cause significant morbidity and mortality worldwide (Tota and others 2011; Forman and others 2012). Of all hrHPVs, HPV16 is by far the most common genotype accounting for ∼50% of cervical cancers and 90% of HPV-related head and neck squamous cell carcinomas (HNSCCs) (Chen and others 2005; Ragin and Taioli 2007). While prophylactic HPV vaccines have demonstrated effective prevention of high-grade cervical lesions associated with hrHPV types 16 and 18 (Schiller and others 2012), widespread HPV vaccination remains poor. For example, the percentage of girls in the United States completing the recommended vaccine schedule was only 37.6% in 2013 (Stokley and others 2014). Additionally, these vaccines provide no protection against disease for the hundreds of millions of people who are already infected with HPV. Notably in the United States, the number of HPV-positive oropharyngeal cancers are expected to rise and will surpass the annual number of cervical cancers by year 2020 (Chaturvedi and others 2011). Hence, a significant number of individuals are still at risk for developing HPV-related cancer as they are already infected, have not been vaccinated, or will become infected by an HPV type not covered in the current bivalent and quadrivalent vaccines, emphasizing the need to expand HPV-related cancer prophylaxis strategies.

While genital hrHPV infections are very common, more than 1 of 7 hrHPV-infected women cannot initiate an effective immune response against the virus, and among those who do, clearance is slow and generally takes more than 1 year (Frazer 1996; Giuliano and others 2002; Stanley and others 2007). To persist in this manner, HPV has evolved mechanisms to escape immune detection, and this persistence is the major risk factor for developing HPV-induced cancers.

In our previous work, we have demonstrated that HPV-mediated suppression of Langerhans cell (LC) immune function is a central mechanism by which HPV evades immune detection (Fausch and others 2005b; Da Silva and others 2014; Woodham and others 2014). This effect is specific to LCs as dendritic cells (DCs) are differentially targeted and thus are activated by HPV (Fausch and others 2003). LCs are the primary antigen-presenting cells (APCs) that encounter the virus in the epithelial and mucosal layers (Merad and others 2008) and are hence responsible for initiating immune responses against HPV. Upon proper pathogenic stimulation, immature LCs undergo maturating changes, including the initiation of signaling cascades, increased expression of antigen presentation and costimulatory molecules, and the release of proinflammatory cytokines. These activated LCs then travel to lymph nodes through chemokine-directed migration and interact with T cells to initiate an adaptive immune response [reviewed in Malissen and others (2014)]. Although LCs exposed to HPV16 are able to take up and present antigen, they do not become functionally mature, exhibit dysregulated cell signaling, and ultimately HPV16-suppressed LCs are unable to activate HPV-specific effector T cells (Fausch and others 2002, 2005b). The suppressive effect is not limited to HPV16, but has also been shown for several other hrHPV genotypes (Da Silva and others 2014). Thus, reversal of hrHPV-mediated suppression of LC immune function represents an important therapeutic target. Identifying immune-modulating agents that reverse this suppression will pave the way for clinical trials to treat persistent HPV infection.

The immune-modulating therapeutic, IRX-2, is a cytokine-based primary cell-derived biologic consisting of multiple well-defined active cytokine components, including interleukin (IL)-1β, IL-2, IL-6, IL-8, tumor necrosis factor alpha (TNFα), granulocyte-macrophage colony-stimulating factor (GM-CSF), and interferon gamma (IFNγ) (Freeman and others 2011), and has recently been used in early stage clinical trials as an immune-enhancing agent for HNSCC patients (Freeman and others 2011; Wolf and others 2011; Berinstein and others 2012; Whiteside and others 2012). In a Phase II clinical trial, locoregional injection of IRX-2 increased activated T-cell infiltration into tumors, which correlated with increased overall survival (Berinstein and others 2012). These promising results may be related to the observation that IRX-2 has been shown to enhance antigen presentation by DCs (Naylor and Hadden 2003; Egan and others 2007; Schilling and others 2013). Specifically, it was demonstrated that monocyte-derived DCs from HNSCC patients matured with IRX-2 displayed increased antigen presentation and costimulatory molecules, secreted the T helper 1 (Th1)/cellular immune response promoting cytokine IL-12p70, and could initiate an adaptive T-cell response (Schilling and others 2013).

As a result of synergy between different cytokines and their actions on multiple cell types, IRX-2 has also been shown to protect T cells from apoptosis (Czystowska and others 2009), increase the T helper-to-T suppressor ratio (Schilling and others 2012b), enhance natural killer (NK) cell function (Schilling and others 2012a), and enhance *in vivo* response to vaccine constructs (Naylor and others 2010; Berinstein and others 2011). These prior *in vitro* and *in vivo* studies suggest that IRX-2 is a promising immunomodulator in a cancer or precancerous setting. However, it has never been shown to induce antiviral activity in the context of HPV infection or HPV-mediated suppression of APC function. Therefore, in the current study, we investigated the hypothesis that IRX-2 would be effective in enhancing the APC activity of LCs by activating HPV16-exposed LCs *in vitro* and potentially reversing HPV-mediated suppression of LC immune function.

## Materials and Methods

### IRX-2 production

IRX-2 is a primary cell-derived biologic with multiple well-defined active cytokines (IRX Therapeutics, New York, NY), which include IL-1β, IL-2, IL-6, IL-8, TNFα, GM-CSF, and IFNγ (Freeman and others 2011). IRX-2 is produced in a scalable, current Good Manufacturing Practices (cGMP) process by inducing proinflammatory cytokine secretion of human peripheral blood mononuclear cells (PBMCs) with phytohemagglutinin A (PHA). The PHA is removed before supernatant collection, which is then subjected to ion exchange and nanofiltration. Stringent quality control, including bioassay and ELISA determination of cytokine levels, assures the consistency of the components in IRX-2. Safety testing for sterility, DNA, mycoplasma, and endotoxin and testing for HIV, CMV, and EBV are also part of the process. IRX-2 dosing is based on IL-2 content. Several lots of IRX-2 were used over the course of these studies, and the average levels of defined cytokines in the lots used were as follows: IL-2 (6.3 ng/mL); IL-1β (0.8 ng/mL); IFNγ (2.4 ng/mL); TNFα (4.0 ng/mL); and IL-6 (1.4 ng/mL). Studies have demonstrated the consistency of the biological activity among lots both *in vitro* and *in vivo* (Naylor and Hadden 2003; Egan and others 2007; Czystowska and others 2009; Naylor and others 2010; Berinstein and others 2011; Whiteside and others 2012). The IRX-2 manufacturing process is approved by the U.S. Food and Drug Administration for Phase I–III clinical testing.

### Antibodies and reagents

HLA-ABC FITC (MHC I), HLA-DP, DQ, DR-FITC (MHC II), CD80 FITC, CD86 FITC, CD83 PE, CD1a PE, CD14 PE, Langerin PE, E-cadherin PE, CD8-FITC, CD3-PE-Cy5, and CCR7 PE were purchased from BD Biosciences (San Jose, CA). CD40 PE, purified rat IgG2a, goat anti-rat IgG PE, mouse IgG1 FITC, and mouse IgG1 PE were purchased from Biolegend (San Diego, CA). Recombinant human (rhu)-CCL21 was purchased from R&D Systems (Minneapolis, MN); rhu-GM-CSF was purchased from Berlex (Seattle, WA); and rhu-transforming growth factor-β1 (TGFβ1) and rhu-IL-4 were purchased from Biosource (Carlsbad, CA).

### HPV16 virus-like particles

HPV16 virus-like particles (VLPs) consisting of the 2 self-assembling capsid proteins responsible for HPV virion assembly and viral genome packaging (L1 and L2, respectively) were produced in insect cells and purified as previously described (Kirnbauer and others 1992). HPV16 L1L2 VLPs are highly immunogenic nonreplicative structures that mimic their virus counterparts in morphology, immunogenicity, and immunosuppression of LCs (Kirnbauer and others 1992, 1993; Breitburd and others 1995; Fahey and others 2009a). Endotoxin levels in VLP preparations were found to be below 0.06 EU using an E-toxate kit (Sigma-Aldrich, Carlsbad, CA). Chimeric HPV16 L1L2 VLPs containing the E7 protein (HPV cVLP) were produced as previously described (Greenstone and others 1998). Chimeric HPV cVLPs were used for *in vitro* immunization experiments in this study to analyze induction of HPV16 E7-specific T cells by LCs. These cVLPs contain a fusion protein of L2-E7, which encapsidates the E7 protein inside the VLPs (Greenstone and others 1998).

### Generation of human LCs and HLA typing

Monocyte-derived primary LCs were generated by plastic adherence of monocytes from commercially obtained HLA-A*0201^+^ PBMCs to culture flasks as previously described (Fahey and others 2009b). Briefly, cryopreserved PBMCs were thawed and washed with RPMI 1640, containing 10 mM sodium pyruvate, 10 mM nonessential amino acids, 50 μg/mL kanamycin, and 10% heat-inactivated fetal bovine serum (referred to as complete medium). Adherent monocytes were cultured in complete medium supplemented with rhu-GM-CSF (1,000 U/mL), rhu-IL-4 (1,000 U/mL), and rhu-TGFβ (10 ng/mL) for 7 days. Cytokines were replenished twice during the differentiation period. These immature LCs are typically Langerin^+^ E-cadherin^+^ MHC class II^+^ CD1a^+^ CD11c^+^ CD86^−^ CD83^−^ as determined by flow cytometry. The University of Southern California's Institutional Review Board approved all studies.

### LC activation and flow cytometry phenotype analysis

LCs were treated with HPV16 VLPs before stimulation, and surface markers were detected by flow cytometry as previously described (Woodham and others 2015). Briefly, 10^6^ LCs were treated with 10 μg HPV16 L1L2 VLPs in 1 mL phosphate-buffered saline for 1 h at 37°C or left untreated. LCs were then transferred to 6-well plates (5 × 10^5^ LC/well) in 3 mL of complete medium. After 6 h, the cells were left untreated or treated with IRX-2 at the indicated dilutions. Recombinant human CD40 ligand (R&D Systems) was used as a positive control to confirm LC responsiveness. After 72 h, the cells were collected and stained for surface Langerin, E-cadherin, major histocompatibility complex (MHC) I, MHC II, CD80, CD83, CD86, CD1a, CD40, CCR7, or isotype controls, and fluorescence intensity was measured using flow cytometry.

### Cytokine and chemokine analysis

Supernatants from 72-h cultures were collected from untreated LCs or LCs treated with HPV16 and/or IRX-2 as described above and assayed in triplicate for secreted cytokines and chemokines using the Bio-Plex Suspension Array System (Bio-Rad, Hercules, CA) (Woodham and others 2015), which allows for several Th1-associated inflammatory and chemoattractant analytes to be assayed at once. Cytokines and chemokines analyzed included IFNα, IFNγ, IL-1β, IL-6, IL-8, IL-10, IL-12p40/p70, IFNγ inducible protein 10 (IP-10), TNFα, monocyte chemoattractant protein 1 (MCP-1), macrophage inflammatory protein (MIP)-1α, MIP-1β, monokine induced by γ-interferon (MIG), and RANTES, using a custom MilliPlex MAP Human Cytokine/Chemokine Panel as per the manufacturer's instructions (Millipore, Billerica, MA). Cytokines in IRX-2 alone were measured to discriminate levels of cytokines produced from LCs from cytokines present in the input IRX-2.

### *In vitro* migration of LCs

Chemokine-directed migration of LCs toward CCL21 was carried out using 24-well Transwell plates with 5-μm pore size polycarbonate filters (Costar, Cambridge, MA) using 250 ng/mL CCL21 in the lower chamber or complete medium alone as a control for spontaneous migration, as previously described (Fahey and others 2009a; Yan and others 2015). Briefly, 2 × 10^5^ untreated LCs or LCs treated with HPV VLPs and/or IRX-2, as indicated in the activation assay above, were added to upper chamber wells in triplicate and incubated for 4 h at 37°C, 5% CO_2_. After 4 h, lower chamber migrated cells were collected and counted using a Z1 Beckman Coulter particle counter.

### Mixed lymphocyte reaction assay

The mixed leukocyte reaction (MLR) assay was performed as described previously (Rudolf and others 2001; Fausch and others 2002; Muul and others 2008). Briefly, HLA-A*0201 LCs were treated (or not) with HPV VLPs and/or IRX-2 and cocultured with untreated allogeneic HLA-mismatched (non-HLA-A2) CD4^+^ and CD8^+^ T cells isolated from different donor PBMCs by negative magnetic separation (Miltenyi Biotec, San Diego, CA). Responder (R) T cells and irradiated stimulator (S) LCs were cultured at an R:S ratio of 20:1 in a 96-well round bottom plate in replicates of 6 per treatment for 5 days. T cells and LCs, each cultured alone, and T cells cultured with autologous PBMCs served as negative controls, while PHA-treated T cells served as a positive control. Five days later, ^3^H-thymidine was added, and after an additional 18 h, radioactive ^3^H-thymidine-pulsed cells were harvested and radioactivity counted on a TopCount microplate liquid scintillation counter to quantify cell proliferation (PerkinElmer, Waltham, MA). In separate experiments, purified T cells were cultured with IRX-2 alone to determine the direct effect of the reagent on T-cell proliferation.

### *In vitro* immunization assay with HPV E7

Autologous T cells and LCs from HLA-A*0201^+^ donors were cocultured *in vitro* for 4 weeks to elicit primary CD8^+^ T-cell responses against HPV16 E7 using our previously protocol (Yan and others 2015). Specifically, LCs were left untreated or exposed to HPV16-L1/L2-E7 cVLPs, then left untreated or treated with IRX-2. Magnetically isolated, untouched, naïve autologous CD8^+^ T cells were cocultured with irradiated LCs at a 20:1 (R:S) ratio for 7 days at 37°C. Cultures were restimulated with untreated or treated LCs at days 7, 14, and 21. After 28 days, T cells were harvested and tested for E7 peptide-specific IFNγ production by ELISpot as a measurement of HPV16-specific CD8^+^ T-cell responses.

### IFNγ ELISpot assay

The ELISpot assay was performed following established laboratory protocols (Yan and others 2015). Specifically, 96-well multiscreen HTS plates (Millipore) were coated with 10 μg/mL anti-human IFNγ (clone 1-D1K; Mabtech, Mariemont, OH) overnight, washed, and blocked for 2 h with complete media at 37°C. T cells from the above *in vitro* immunization assay were plated at 2 × 10^5^ cells per well in quadruplicate with or without the HLA-A*0201-restricted HPV16 peptides E7_11–20_, E7_82–90_, or E7_86–93_ for 18 h at 37°C. Wells were washed and plates were incubated with 1 μg/mL biotinylated anti-human IFNγ antibody (clone 7-B6-1; Mabtech) for 2 h, followed by incubation with streptavidin-HRP for 1 h (Sigma-Aldrich). Individual spots were counted after development with 3-amino-9-ethyl-carbazole substrate in 0.05 M sodium acetate buffer (Sigma-Aldrich) using the automated computer-assisted video-imaging KS ELISPOT analysis system (Carl Zeiss, Thornwood, NY). The average number of background spots (wells incubated with control peptide) was subtracted from E7 peptide-stimulated wells to quantify peptide-specific responses.

### Statistical analyses

GraphPad Prism 6 was used for all statistical analyses (San Diego, CA). Significance was determined by a 1-way ANOVA, followed by Tukey's post-test, comparing individual treatments.

## Results

### IRX-2 treatment results in phenotypic activation of LCs

**FIG. 1.**
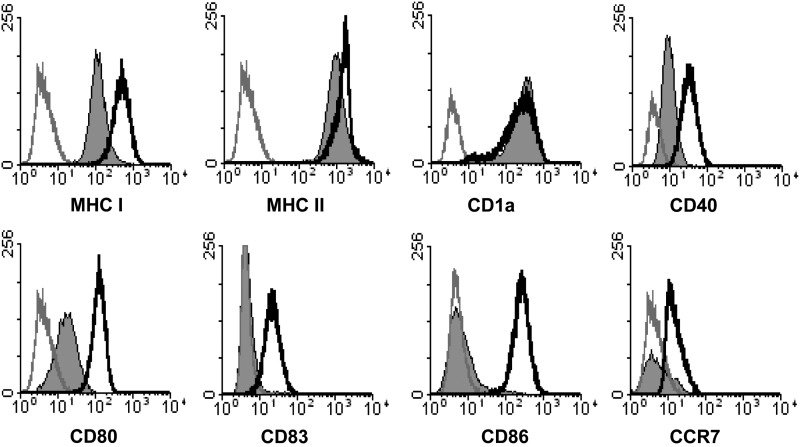
IRX-2 induces phenotypic activation of human Langerhans cells (LCs). LCs were left untreated (*solid gray histograms*) or stimulated with IRX-2 (*unfilled black histograms*) at a 1:2 dilution for 72 h at 37°C. Resultant concentrations of input cytokine mix were 3.2 ng/mL interleukin (IL)-2; 0.4 ng/mL IL-1β; 1.2 ng/mL interferon gamma (IFNγ); 2.0 ng/mL tumor necrosis factor alpha (TNFα); and 0.7 ng/mL IL-6. After the incubation, the cells were analyzed by flow cytometry for the expression of major histocompatibility complex (MHC), costimulatory molecules, maturation markers, and CCR7. Isotype controls are shown as *unfilled gray histograms*. Data are representative of 4 individual donors.

The phenotype of immature LCs derived from monocytes was defined as high expression of MHC class I, class II, and CD1a with low expression of costimulatory molecules and maturation markers, CD40, CD80, CD83, CD86, and CCR7 (Fig. 1, solid gray histograms), similar to what has been shown for LCs isolated from skin *ex vivo* (Flacher and others 2006). Furthermore, studies have shown that *in vitro* derived LCs express the same surface markers as immature epidermal LCs and are an appropriate source for LC functional studies (Geissmann and others 1998; Fausch and others 2003, 2005a; Ginhoux and others 2006).

Immature LCs were incubated with a 1:2 dilution of IRX-2 with resultant concentrations of 3.2 ng/mL IL-2; 0.4 ng/mL IL-1β; 1.2 ng/mL IFNγ; 2.0 ng/mL TNFα; and 0.7 ng/mL IL-6, and the same dilution was used throughout this study. Subsequently, a large increase in cell surface expression of all the aforementioned maturation markers was observed (Fig. 1, unfilled black histograms), indicating that IRX-2 was able to significantly induce an activated LC phenotype. The expression level of CD1a did not change as expression of this alternative antigen-presenting molecule was already high. Additionally, typical LC phenotypic marker expression, such as that of Langerin and E-cadherin, was unchanged after IRX-2 treatment compared with that of untreated LCs (data not shown). The increase in activation-associated surface markers was verified with 2 separate manufactured lots of IRX-2 to demonstrate consistency of IRX-2 activity between lots, and the activity was shown to be dose dependent when used at a 1:2 and 1:3 dilution (3.2 > 2.1 > 0 ng/mL of IL-2 equivalents) (Supplementary Fig. S1; Supplementary Data are available online at www.liebertpub.com/jir).

**FIG. 2.**
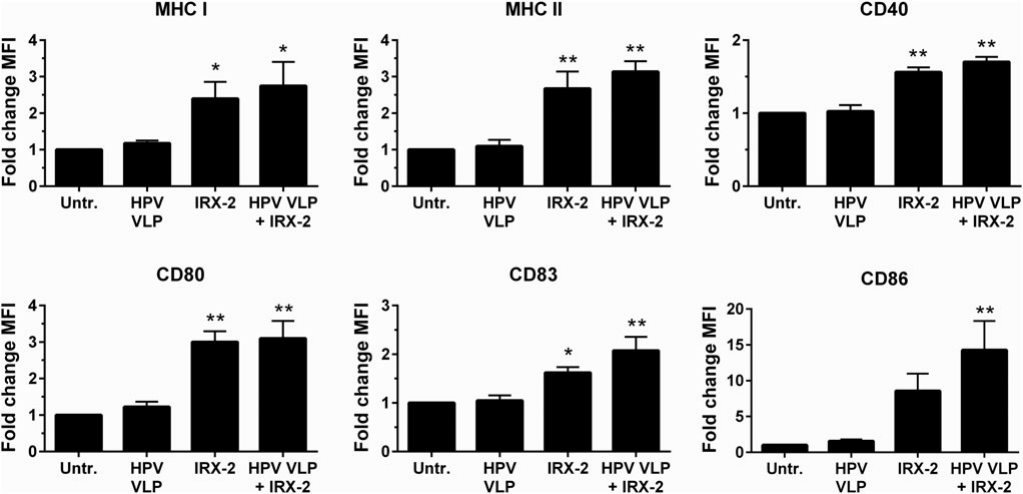
IRX-2 reversal of human papillomavirus (HPV) inhibition of LC activation. LCs were exposed to HPV16 virus-like particles (VLPs) for 6 h. IRX-2 (1:2 dilution) or media were then added for 72 h at 37°C. After incubation, the cells were analyzed by flow cytometry for the expression of the indicated surface markers associated with activation. Shown is the fold change in mean fluorescence intensity ± standard error of the mean (SEM) of antibody staining for each marker compared with baseline unactivated LCs. Data represent the mean of 4 individual donors. *P < 0.05, **P < 0.01 compared with untreated in each graph.

Our previous studies have shown that monocyte-derived DCs become activated when exposed to HPV16, whereas LCs do not show phenotypic or functional activation (Fausch and others 2002, 2003). Therefore, the ability of IRX-2 to induce LC activation in the presence of HPV16 was examined. Exposure of LCs to HPV16 VLPs did not upregulate MHCs and costimulatory molecules compared with unexposed LCs (Fig. 2), similar to our previous findings (Fausch and others 2002), In contrast, IRX-2 treatment of HPV16-exposed LCs caused a significant upregulation of antigen presentation and costimulatory molecules compared with untreated controls in all 4 donors tested (Fig. 2). Importantly, HPV exposure did not result in reduced IRX-2 activating potency. These results indicate that IRX-2 is able to induce phenotypic activation of LCs, despite the presence of HPV16, suggesting a potential reversal of the HPV-mediated suppression of LC immune function.

### IRX-2 induces inflammatory cytokine and chemokine secretion from HPV16-exposed LCs

**FIG. 3.**
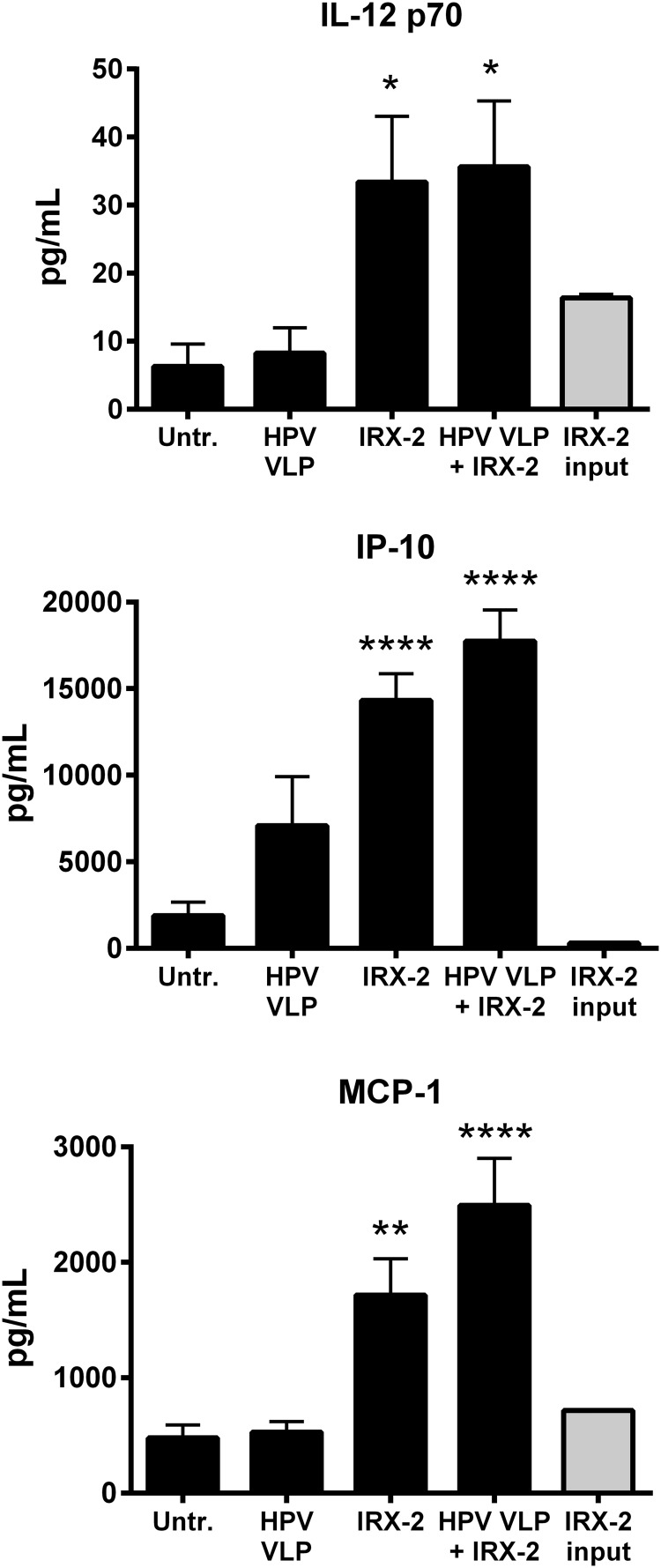
IRX-2 induces cytokine and chemokine secretion by human LCs exposed to HPV16 VLPs. LCs were exposed to HPV16 VLPs for 6 h at 37°C, then stimulated IRX-2 at a 1:2 dilution for 72 h at 37°C. After the incubation, supernatants were collected and assayed for IL-12p70, inducible protein 10 (IP-10), and monocyte chemoattractant protein 1 (MCP-1) by multiplexing ELISA in triplicate per healthy donor. Control LCs were left untreated, treated with HPV16 VLPs alone, or treated with IRX-2 alone. Shown is the concentration of secreted cytokine or chemokine with each LC treatment (*black bars*) and the concentration of cytokine or chemokine present in the diluted IRX-2 reagent (*gray bar*). Data represent the mean ± SEM of 4 individual donors. ****P < 0.0001, **P < 0.01 compared with untreated control.

Activated LCs produce Th1-associated cytokines to assist in priming CD8^+^ T cells against viral antigens and produce chemokines to recruit innate immune cells to participate in the eradication of virus-infected cells. Therefore, the ability of HPV16-exposed LCs to secrete a variety of cytokines and chemokines with or without IRX-2 treatment was examined. Because IRX-2 is a naturally produced cytokine mixture derived from PBMCs, the input amount of IRX-2 was measured to identify the source of the measured cytokines. Our results show that treatment of HPV16-exposed LCs with IRX-2 resulted in significantly increased secretion of IL-12p70, IP-10, and MCP-1 compared with untreated or HPV-only-treated LCs (Fig. 3). Other cytokines measured (IFNα, IFNγ, IL-1β, IL-6, IL-8, IL-10, TNFα, MIP-1α, MIP-1β, MIG, and RANTES) were not found in higher levels than the input amount of cytokines contained in IRX-2 (data not shown). Notably, HPV16 exposure did not block the ability of IRX-2 to induce the secretion of cytokines, demonstrating that HPV16-exposed LCs are able to secrete cytokines that could activate and attract T cells to the site of antigen priming after treatment with IRX-2.

### IRX-2 treatment results in CCL21-directed migration of HPV16-exposed LCs

**FIG. 4.**
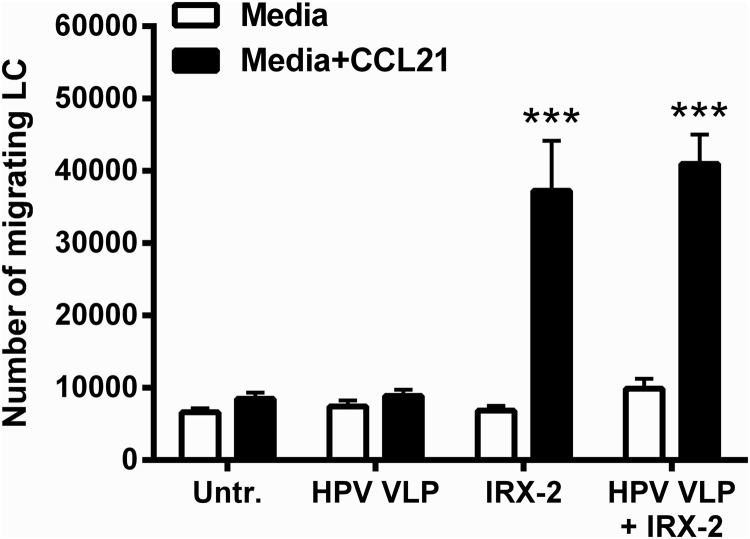
IRX-2 induces migration of human LCs exposed to HPV16 VLPs. LCs were exposed to HPV16 VLPs for 6 h at 37°C, then stimulated IRX-2 at a 1:2 dilution for 72 h at 37°C. After the incubation, the cells were analyzed in an *in vitro* transwell migration assay in triplicate per healthy donor. Control LCs were left untreated, treated with HPV16 VLPs alone, or treated with IRX-2 alone. Shown is the number of LCs that chemotax spontaneously to media alone and the number of LCs that chemotax toward the chemokine, CCL21. Data represent the mean ± SEM of 4 individual donors. ***P < 0.001 compared with untreated control.

Chemokine-directed migration of periphery activated LCs to regional lymph nodes is required for successful naïve T-cell interaction (Randolph and others 2008). Immature LCs upregulate expression of CCR7 upon activation to migrate to the lymph nodes where the CCR7 chemokine ligand, CCL21, is expressed. This necessary hallmark of LC activation does not occur in the presence of HPVs, resulting in an inability of LCs to migrate to lymphoid tissues after picking up HPV antigens (Fausch and others 2002). As an *in vitro* evaluation of LC migratory capacity, a transwell chemotaxis assay to CCL21 was performed to measure LC migration after exposure to HPV16, followed by treatment with IRX-2, since LC treatment with IRX-2 alone was shown to induce CCR7 expression (Fig. 1, bottom right panel). Treatment of LCs with IRX-2 alone or IRX-2 treatment after HPV16 exposure resulted in a significant increase in migration toward CCL21 compared with untreated LCs or LCs exposed to HPV16 alone (Fig. 4), suggesting that IRX-2-treated LCs acquire the functional ability to migrate.

### IRX-2 enhances T-cell stimulatory capacity of HPV16-exposed LCs and induces HPV16-specific CD8^+^ T-cell responses

**FIG. 5.**
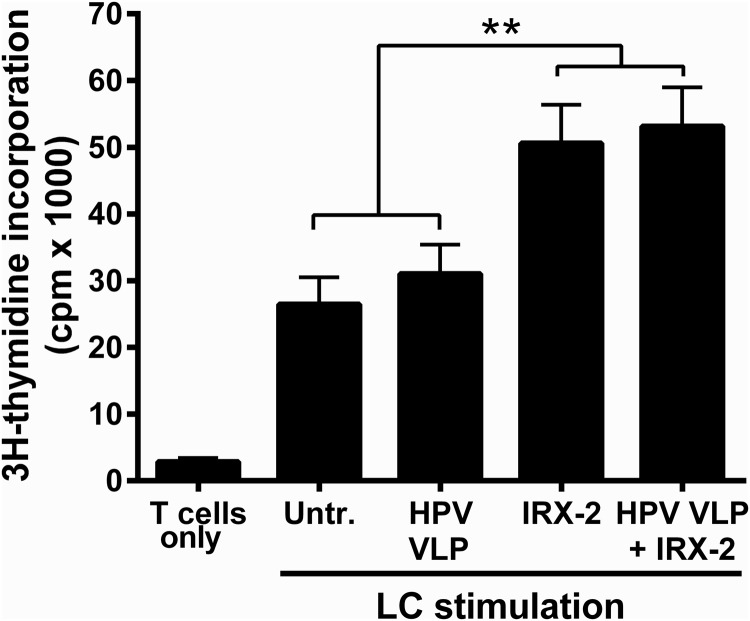
Human LCs exposed to IRX-2 are superior in stimulating allogeneic T cells even in the presence of HPVs. LCs were exposed to HPV16 VLPs for 6 h at 37°C, then stimulated with IRX-2 (1:2 dilution) for 72 h at 37°C. Control LCs were left untreated, treated with HPV16 VLPs alone, or treated with IRX-2 alone. LCs were collected, washed, irradiated, and cocultured for 7 days with purified allogeneic human CD3^+^ T cells at a 1:20 ratio in a mixed lymphocyte assay. On day 6, cells were pulsed with ^3^H-thymidine for 18 h. On day 7, cells were harvested onto filter plates and incorporated thymidine measured on a radioactive scintillation counter. Data represent the mean counts per minute (cpm) ± SEM of 4 individual donors. **P < 0.01 compared with untreated control.

Activated LCs can robustly stimulate the proliferation of T cells (Stingl and others 1978) and therefore an *in vitro* MLR assay was used to determine whether IRX-2-treated LCs pre-exposed to HPV16 can stimulate naïve T-cell proliferation. An MLR is a traditional immunological assay that measures the level of reactivity between T cells from 1 donor and APCs from another HLA-mismatched donor by demonstrating increased T-cell proliferation to allogeneic LCs due to upregulated expression of mismatched MHCs. Untreated LCs, HPV16-exposed LCs, or LCs treated with IRX-2 with or without HPV16 pre-exposure were cocultured with allogeneic donor T cells. LCs treated with IRX-2 alone and following HPV16 exposure demonstrated a significant enhancement of T-cell stimulatory capacity compared with untreated LCs or HPV16-treated LCs, measured by the increase in T-cell proliferation (Fig. 5). Additionally, we found that direct treatment of T cells with IRX-2 induced proliferation at levels lower than those seen when LCs were treated with IRX-2 before coculture with T cells (Supplementary Fig. S2). This suggests that while IRX-2 has the capacity to directly induce T-cell proliferation (likely due to the presence of IL-2), the coculture of T cells with IRX-2-activated LCs is superior in inducing T-cell proliferation since IRX-2 was washed away before all cocultures.

In addition to stimulating naïve allogeneic T-cell proliferation, activated LCs should also induce antigen-specific T-cell responses. As CD8^+^ cytotoxic T cells are essential to the response for clearing viral infections and killing tumor cells, the ability of LCs to stimulate an HPV16 antigen-specific CD8^+^ T-cell response would serve as strong evidence that HPV16 suppression of LC activation can be overcome. Therefore, to analyze the ability of IRX-2-treated LCs to induce HPV16-specific CD8^+^ T cells, an *in vitro* immunization assay, followed by an IFNγ ELISpot, was performed to measure peptide-specific secretion of IFNγ, a signature cytokine for activated antigen-specific T-cell effector function. HPV16 chimeric VLPs were utilized in these experiments, incorporating a fusion protein of L2-E7, which encapsidates the E7 protein within the cVLPs (Greenstone and others 1998). These cVLPs enter LCs as native HPV virions would and allow E7 to be processed as a viral antigen and loaded on MHC molecules (ie, HLA-A*02) through antigen cross-presentation pathways. Three defined HLA-A*0201 binding E7 epitopes were used to detect the induction of reactive HPV16 E7-specific CD8^+^ T cells (Kast and others 1994).

**FIG. 6.**
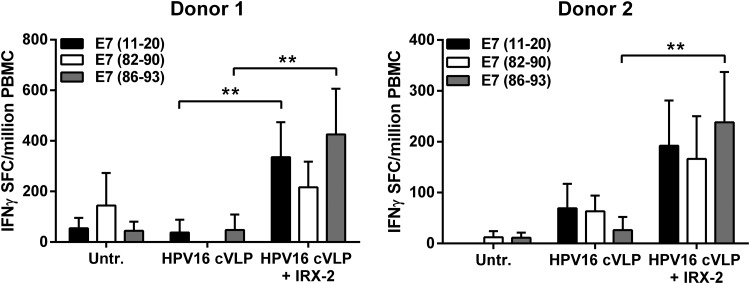
Induction of HPV16 epitope-specific CD8^+^ T cells by IRX-2 through the activation of LCs exposed to HPV16 cVLP. LCs from an HLA-A* 0201-positive blood donor were incubated with media alone (Untr.), with HPV16 cVLP alone, or with HPV16 cVLP and IRX-2 (1:2 dilution). The treated LCs were incubated with purified autologous CD8^+^ lymphocytes and restimulated weekly for 4 weeks. Responder cells were analyzed on day 28 for IFNγ production in an ELISpot assay against the indicated E7 peptides. Data are expressed as the mean IFNγ spot-forming cells (SFCs) per million peripheral blood mononuclear cells (PBMCs) ± standard deviation of replicate wells. Two individual donors are shown. **P < 0.01, comparing individual peptide-specific responses in IRX-2-treated HPV16 cVLP-exposed LCs versus HPV16 cVLP-exposed LCs alone. CD8^+^ T-cell responses from donor 2 for all 3 peptides in the IRX-treated group were significant compared with untreated LCs (*P* < 0.05).

Two separate HLA-A*0201^+^ donor LCs, which were exposed to HPV16 cVLP and subsequently treated with IRX-2, were able to significantly induce IFNγ-secreting E7_86–93_ peptide-specific CD8^+^ T cells when compared with untreated LCs or LCs exposed to HPV16 cVLPs alone (Fig. 6). IRX-2-treated LCs from donor 1 significantly increased E7_11–20_ peptide-specific CD8^+^ T cells in addition to the E7_86–93_ peptide-specific response. IRX-2-treated LCs from donor 2 induced significant CD8^+^ T-cell responses to all 3 peptides compared with untreated LCs (*P* < 0.05). Although the resulting spot numbers against some individual HPV peptides were low, this is expected from a *de novo* induction of antigen-specific T cells *in vitro* given that the response to E7 was polyclonal. T cells alone (without LC coculture) did not expand or survive the duration of the experiment. Taken together, these results demonstrate that LCs exposed to HPV16 before IRX-2 treatment *in vitro* became functionally active APCs capable of inducing T-cell proliferation and activating HPV16-specific CD8^+^ T-cell responses.

## Discussion

It has been reported that HPV16 capsids can stimulate the activation of human DCs (Lenz and others 2001; Rudolf and others 2001), providing evidence that HPV16 can induce the maturation of certain APC subsets. In contrast, the suppression of LC immune function by HPVs is manifested by dysregulation of the PI3K-Akt signaling axis, resulting in the lack of phenotypic and functional activation (Fausch and others 2002, 2005b). The inhibitory effects of the virus on LC signaling and subsequent activation occur within 15 min of HPV16 exposure (Fausch and others 2005b). Specifically, our previous work using HPV16 VLPs and either monocyte-derived DCs or LCs demonstrated that activation-associated cell surface markers were increased in DCs exposed to HPV16 VLPs, but not LCs (Fausch and others 2002). Furthermore, our previous data indicate that the interaction of HPV16 and other hrHPV types with LCs results in a suppressive signaling cascade, suggestive of active immune suppression *in vitro* (Fausch and others 2005b; Da Silva and others 2014). More recently, it was demonstrated that mucosal resident LCs in HPV-induced cervical lesions were spherical, lacked dendrites, and secreted the suppressive cytokine, IL-10 (Prata and others 2015). The authors further demonstrated that the number of IL-10-secreting LCs, which were characterized as an immunosuppressive phenotype, and the amount of IL-10 produced in lesions corresponded with severity of histopathology and HPV viral load, providing strong evidence of an active immunosuppressive mechanism employed by HPVs targeting LCs *in vivo*.

In this study, we show that IRX-2 phenotypically and functionally activates LCs, both in the absence and presence of HPV16. The latter is important because it demonstrates that IRX-2 can be used as an immunostimulatory agent to activate LCs that are otherwise presenting HPV antigens in an immature potentially immunosuppressive state. Among the IRX-2-induced phenotypical changes was the upregulation of antigen presentation and costimulatory molecules, resulting in an increased capacity of LCs to present viral antigens. The accompanying increase in CD40, CD80, and CD86 expression provides costimulation for proper T-cell activation. These results are in line with previous studies from our group, demonstrating that HPV-exposed LCs from both healthy donors and women with cervical precancerous lesions caused by persistent hrHPV infection are capable of being functionally activated with TLR3 or TLR8 agonists, which provide a necessary danger signal to stimulate T-cell activation (Fahey and others 2009b; Da Silva and others 2015a, 2015b). Thus, the inflammatory cytokines contained in IRX-2 are able to induce functional LCs in the presence of HPVs, similar to other conventional maturation stimuli such as TLR agonists.

Although it is a cytokine mixture itself, IRX-2 induced the secretion of IL-12p70, IP-10, and MCP-1 from LCs, of which the latter are involved in the activation and chemoattraction of Th1 and other immune cells (Dufour and others 2002; Deshmane and others 2009). Importantly, the induced levels were significantly higher than the input levels contained in IRX-2. This mix of cytokines provides an immune-stimulating environment and correlates with the mixture of cytokines induced by treatment of HPV16-exposed LCs with the TLR3 agonist Poly-I:C (Da Silva and others 2015a).

As the primary member of the IL-12 pleiotropic family of cytokines, IL-12p70 is produced by DCs and DC subsets (ie, LC) in response to pathogens, including viruses, and IL-12p70 is the signature IFNγ-inducing cytokine (Trinchieri 2003). IFNγ is the canonical Th1 cytokine that is crucial for a cellular immune response as it controls the differentiation of naïve CD4^+^ T cells into Th1 effectors, which mediate cellular immunity against viral infections (Schoenborn and Wilson 2007). Additionally, IL-12 promotes T-cell proliferation and enhances cytotoxicity (Trinchieri 2003). Interestingly, the ratio of IL-12 to the suppressive cytokine IL-10 has been suggested to predict the *in vivo* potency of DCs, and this ratio was swayed in favor of IL-12 upon IRX-2 treatment of DCs (Schilling and others 2013). In light of recent evidence that HPV induces an immunosuppressive phenotype of LC that produces IL-10 *in vivo* (Prata and others 2015) and the current results reported herein that IRX-2 increases IL-12 secretion by LCs, it is reasonable to envision a mechanism by which IRX-2 could positively influence the IL-12/IL-10 ratio to promote HPV clearance.

IRX-2 is a primary cell-derived biologic comprising consistent quantities of multiple cytokines and chemokines that reflect physiologic concentrations (Hadden and others 1994; Egan and others 2007; Freeman and others 2011). This makes the effective cytokine concentrations in IRX-2 up to 60 times lower than those of recombinant cytokines used in *ex vivo* DC maturation (Reis e Sousa 2006; Muthuswamy and others 2010) and lower still than those used in high-dose systemic cytokine therapies (Rosenberg and others 1987, 1994). While cytokine-based treatments have not been widely investigated in cervical precancerous lesions or cancer, IFN treatments (primarily α and β) for HPV type 6- and 11-induced genital warts have been widely used (Yliskoski and others 1991; Olmos and others 1994; Friedman-Kien 1995; Beutner and Ferenczy 1997; Syed and Ahmadpour 1998), and a systematic review demonstrated that local administration was more effective than systemic use (Yang and others 2009). However, a single cytokine may have limited mechanisms of action and may require higher dosages. Therefore, the physiologic levels of various cytokines in IRX-2 can be administered locally (rather than systemically) into patients and synergize together to avoid significant high-dose cytokine-associated toxicities associated with administration of a single recombinant cytokine.

Although the IRX-2 reagent was diluted for the *in vitro* studies described here to maintain the LCs in culture medium, *in vivo* it would be injected directly into the lesion sites undiluted in its final formulation based on standardization of the IL-2 cytokine concentration. Injected cytokines would be available to immediately bind receptors expressed on LCs and other cells in the tissue. Unbound cytokines would persist for an unknown, but most likely short, duration before draining to the local lymph nodes, where further interactions between LCs and T cells would promote T-cell priming. At a 1:3 *in vitro* dilution of IRX-2, LCs were still able to upregulate costimulatory and maturation markers (Supplementary Fig. S1), suggesting that even when the IRX-2 is dispersed throughout the tissue after injection, lower concentrations of the cytokine mixture will still likely induce LC activation. Additionally, long-term persistence of IRX-2 need not be present to cause LC activation since experiments in which IRX-2 was removed from LC cultures after 24 h yielded similar results in LC activation (data not shown).

While the primary mechanism for IRX-2 effectiveness in immunotherapeutics for HPV-cervical cancer is predicted to be through the reversal of HPV-mediated suppression of LC activation, IRX-2 also contains cytokines that are critical mediators of T-cell activation and proliferation, thus providing additional avenues of immune-enhancing activity. Moreover, if IRX-2 is used therapeutically against more established HPV infections, or in high-grade lesions, its effects would be expected to primarily manifest through other mechanisms such as NK and T-cell activation since viral capsid production would be minimal or absent. The ability of IRX-2 to act on both LCs and other immune cells makes it especially attractive as a treatment for HPV-induced cancers since it is this combination of cells that coordinates immune responses against HPV-infected cells.

IRX-2, which is currently produced under cGMP conditions with rigorous release criteria, has not yet been evaluated as a cervical cancer immunotherapeutic in a clinical trial. However, an early IRX-2 biologic, originally called natural cytokine mixture and produced by a similar protocol, has been evaluated for its impact on early stage cervical carcinoma through an investigator-initiated exploratory study (Duenas-Gonzalez and others 2002). In the study, 10 patients with early stage cervical carcinoma were treated with IRX-2 before definitive treatment with either surgery or radiation. IRX-2 was delivered as injections into the 4 quadrants of the cul-de-sac around the tumor. Biopsied samples were compared with the surgical samples, and in the majority of cervical cancer patients, an increase in leukocyte infiltration of the tumors at surgery was noted, and a significant number of patients remained disease free at 30 months. The increase in leukocyte infiltration observed in this pilot study is similar to that seen in patients with HNSCC where IRX-2 increased the presence of activated T cells in the tumor after therapy and this was associated with increased survival (Berinstein and others 2012). Furthermore, due to evidence that other hrHPV genotypes such as HPV18, HPV31, and HPV45 suppress LC activation through a similar mechanism (Da Silva and others 2014), an HPV genotype independent immune-modulating treatment such as IRX-2, shown to be successful against HPV16, is likely to have efficacy against other hrHPV types.

The *in vitro* activation of LCs by the cytokine-based biologic, IRX-2, reported herein merits the use of IRX-2 in future clinical trials to evaluate its ability to generate a potent LC-mediated Th1-associated response and generate effective HPV-specific CD8^+^ T cells *in vivo*. Such trials would follow the immune response of direct IRX-2 injection into the cervix, where it is expected to reverse the immune suppression mediated by HPVs on local LCs. Therefore, due to its cGMP production, good safety profile in HNSCC clinical trials, and current results, the use of IRX-2 to promote LC activation *in vivo* to clear HPV-infected dysplastic cells should be further explored to treat HPV-induced cancers.

## Supplementary Material

Supplemental data

Supplemental data
