# Supplementary material for: Immunostimulatory Activity of the Cytokine-Based Biologic, IRX-2, on Human Papillomavirus-Exposed Langerhans Cells
Source: J Interferon Cytokine Res. 2016 May 1;36(5):291–301. doi: 10.1089/jir.2015.0115 (PMC4854212; doi:10.1089/jir.2015.0115)
Supplement: Supplemental data [file Supp_Fig1.pdf]

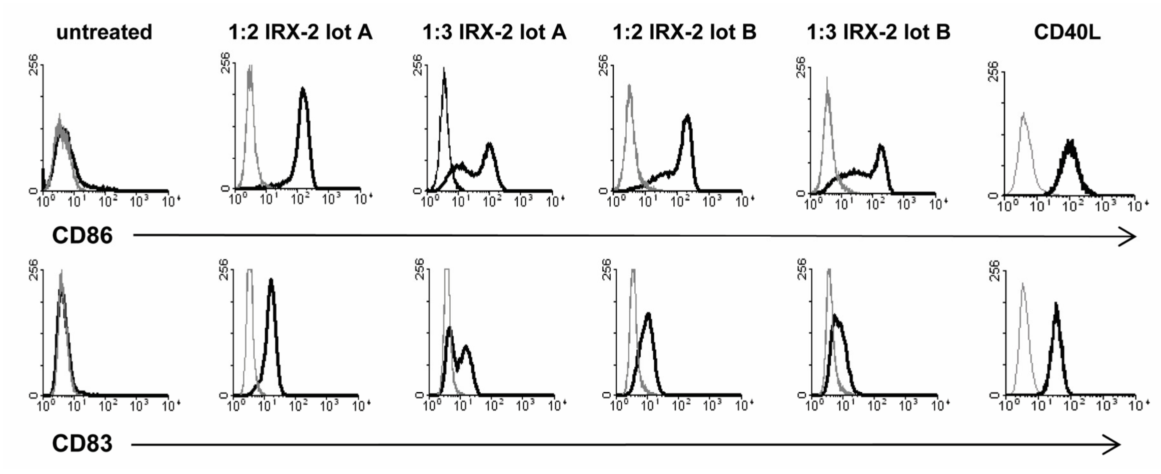

Supplemental Figure 1. Activation and maturation of human Langerhans cells with IRX-2 is dose dependent and independent of the lot of IRX-2. LC were left untreated, stimulated with either of two lots of IRX-2 at a 1:2 or 1:3 dilution, or incubated with 10 μg/mL CD40L for 72 h at 37°C. After incubation the cells were analyzed by flow cytometry for the expression of CD86 and CD83 (thick black lines) or isotype controls (thin gray lines). Data are representative of four independent experiments.
