# Supplementary material for: Immunostimulatory Activity of the Cytokine-Based Biologic, IRX-2, on Human Papillomavirus-Exposed Langerhans Cells
Source: J Interferon Cytokine Res. 2016 May 1;36(5):291–301. doi: 10.1089/jir.2015.0115 (PMC4854212; doi:10.1089/jir.2015.0115)
Supplement: Supplemental data [file Supp_Fig2.pdf]

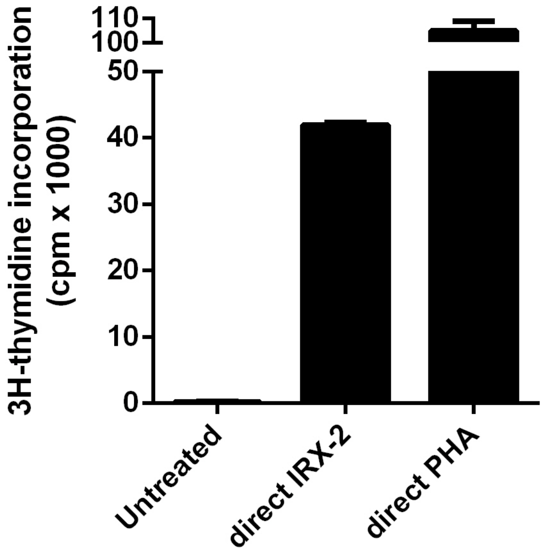

Supplemental Figure 2. T cells were left untreated or were treated with IRX-2 or PHA (positive control) for 72 h at 37°C. After 48 h, cells were pulsed with 3H-thymidine for 18 h. On day 3, cells were harvested onto filter plates and incorporated thymidine measured on a radioactive scintillation counter. Data represent the mean counts per minute (cpm) ± SD.
